# Applicant Demographics and Multiple Mini Interview Performance at a Medical School Over 5 Years

**DOI:** 10.1001/jamanetworkopen.2026.9595

**Published:** 2026-04-22

**Authors:** Trevonne M. Thompson, Yoon Soo Park, Monica Vela, Ara Tekian

**Affiliations:** 1 Department of Emergency Medicine, University of Illinois College of Medicine, Chicago; 2 Department of Medical Education, University of Illinois College of Medicine, Chicago; 3 Department of Medicine, University of Illinois College of Medicine, Chicago

## Abstract

**Importance:**

Multiple mini interview (MMI) is a widely used component of holistic evaluation for medical school admissions; limited studies have evaluated performance of MMI based on applicant sociodemographic characteristics.

**Objective:**

To evaluate differences in MMI performance by medical school applicants of various racial and ethnic groups based on age, gender, and socioeconomic status.

**Design, Setting, and Participants:**

Retrospective cross-sectional study of applicants who participated in MMI from 2020 to 2024 at a large, public medical school in the US.

**Exposure:**

Participation in MMI.

**Main Outcomes and Measures:**

Compared differences in MMI performance based on sociodemographic characteristics by racial and ethnic groups. Performed *t* tests to conduct bivariate comparisons of scores across various groups. Performed multiple linear regression to analyze how independent demographic variables were associated with MMI scores.

**Results:**

There were 3447 applicants who participated in MMI (56 [2%] American Indian or Alaskan Native; 853 [25%] Asian; 650 [19%] Black or African American; 660 [19%] Hispanic or Latino; 31 [1%] Native Hawaiian or Pacific Islander; 1502 [44%] White; 1968 [57%] aged 23 and younger; 1798 [52%] women; and 1198 [35%] with disadvantaged status). The mean (SD) MMI score was 3.77 (0.43) on a scale of 1 to 5 for 3447 applicants. Black or African American (mean [SD] MMI, 3.85 [0.44]; [data]; *P* < .001), compared with other racial groups, and women (mean [SD] MMI, 3.82 [0.41]; [data]; *P* < .001), compared with other gender identities, applicants scored higher. White (mean [SD] MMI, 3.74, [0.43]; [data]; *P* = .001), compared with other racial groups, and disadvantaged (mean [SD] MMI, 3.74 [0.43]; [data]; *P* = .009), compared with not disadvantaged, applicants scored lower. Younger American Indian or Alaskan Native applicants scored higher compared with other racial groups (coefficient [C] = 0.23; SE, 0.11; *P* = .045). Men (C = −0.06; SE, 0.03; *P* = 0.02), compared with other gender identities, and disadvantaged (C = −0.07; SE, 0.03; *P* = .04), compared with not disadvantaged, Asian applicants scored lower. Younger (C = −0.08; SE, 0.03; *P* = .03), compared with older applicants, and men (C = −0.09; SE, 0.04; *P* = .009), compared with other gender identities, Black or African American applicants scored lower. Men (C = −0.97; SE, 0.02; *P* < .001), compared with other gender identities, and disadvantaged (C = −0.68; SE, 0.02; *P* = .007), compared with not disadvantaged, White applicants scored lower. Men Hispanic or Latino applicants (C = −0.11; SE, 0.03; *P* = .002) compared with other ethnicities scored lower.

**Conclusions and Relevance:**

In this study of MMI performance, there were differences in scores by various sociodemographic groups. These results can add to the current understanding of the use of MMI as one tool in the holistic evaluation of medical school applicants.

## Key Points

**Question:**

Does medical school applicant performance on multiple mini interviews (MMI) vary by age, gender, race and ethnicity, and socioeconomic status?

**Findings:**

In this cohort study with 3447 applicants, gender, age, socioeconomic status, and race but not ethnicity were associated with differences in MMI performance. Disaggregation of key demographic groups revealed further differences in MMI performance.

**Meaning:**

These findings suggest that understanding MMI performance may be more complex than reported by prior studies and is worthy of further study.

## Teaser

This cross-sectional study evaluates differences in multiple mini interview (MMI) performance by medical school applicants of various racial and ethnic groups based on age, gender, and socioeconomic status.

## Introduction

The medical school admission process can be considered the most important evaluation procedure conducted by North American medical schools. This assertion is supported by the low attrition rate—less than 3%—of medical students.^1^ Interviews are a valued assessment in the medical school student selection process. Nearly all North American medical schools use some sort of interview as part of their student selection process.^2^

In the early 2000s, faculty at McMaster University developed the multiple mini interview (MMI) in response to the growing concerns about the limitations—such as subjectivity, inconsistency, and bias—of the traditional one-on-one interviews.^3^ The MMI involves several short, timed interview stations focusing on different competencies, skills, or attributes.^3^ Candidates move from one station to the next, encountering different interviewers and scenarios at each.^3^ The structure of the MMI provides a more comprehensive and objective assessment of candidates by using multiple stations, each focusing on different skills, qualities, and decision-making processes.^3^ The MMI format has been shown to be a reliable method of interview evaluation that may reduce the influence of bias on the assessment of medical school applicants.^4^ While MMIs are currently being used in medical college admissions in addition to, or in replacement of, traditional interview formats, few studies have assessed the influence various demographic characteristics may have on MMI performance.^5,6,7^

Previous studies have shown that older age and female gender are associated with higher MMI performance, while lower socioeconomic status or disadvantaged status was associated with lower MMI performance.^5,6^ Studies have also shown no difference in MMI performance between applicants categorized as underrepresented in medicine compared with those categorized as not underrepresented in medicine; however, these studies did not assess MMI performance by the distinct racial and ethnic groups themselves—underrepresented racial and ethnic groups were considered as a combined cohort.^5,6,7^ Previous studies have not thoroughly assessed MMI performance by specific, individual racial and ethnic groups or whether there are differences in performance based on racial and ethnic groups and other characteristics such as age, gender, and sociodemographic status. Knowing that some sociodemographic features, but not others, were associated with differences in MMI performance in studies where many applicant characteristics were aggregated, we sought to evaluate disaggregated applicant characteristics in a large and diverse cohort. We hypothesized that there are differences in MMI performance by applicants to medical school of various racial and ethnic groups based on age, gender, and socioeconomic status.

## Methods

### Setting

The University of Illinois College of Medicine (UI COM) has used MMIs as part of its admissions process since the 2020 admissions cycle. The MMI stations used are adapted from the MMI format developed at McMaster University by Eva et al^3^ and marketed by ProspectHR MMI. The UI COM admissions team conducts 7 MMI stations for each applicant who is interviewed. The same 7 stations are used throughout an admissions cycle. The unique MMI stations are not reused in subsequent admissions cycles; new MMI stations are used each admissions cycle. The MMI stations are all in the format of a written prompt that requires a verbal response. There are no skills stations. The MMI stations are blueprinted to evaluate self-awareness, ethical and moral judgment, responsibility, and empathy. An individual station may assess more than 1 of each of these attributes. Each of the MMI stations is graded on a scale of 1 to 5 (1 = unsuitable to 5 = outstanding) by assessors who are trained to conduct the MMI stations. The MMI stations are blueprinted to the same competency domains—self-awareness, ethical and moral judgement, responsibility, and empathy—over the assessment period and have not changed during the duration of this study. The blueprinting aspect of the MMI allows comparability of MMI scores for admissions decisions annually.

The institutional review board at the University of Illinois Chicago approved this study and determined it to be exempt from human participants research, waiving the requirement for informed consent. The study followed the Strengthening the Reporting of Observational Studies in Epidemiology (STROBE) reporting guideline for cohort studies.

### Data

The MMI scores for the 7 stations for each applicant are collected and then averaged to inform admissions decisions. All scores are recorded on an electronic platform. The electronic platform also contains demographic information for each applicant as provided by the American Medical College Application Service (AMCAS). The demographic information includes race, ethnicity, age, gender, and markers of disadvantaged status. The categories race, ethnicity, age, and gender are self-reported by the applicant on the AMCAS application. For this study, we defined disadvantaged status as applicants identified in their AMCAS application by the first-generation-college-student indicator,^8^ the disadvantaged socioeconomic status indicator,^9^ or the use of the Association of American Medical Colleges fee assistance program.^10^ We included all applicants who interviewed with UI COM during the 2020 to 2024 admissions cycles. We exported the deidentified demographic data and MMI scores for each interviewed applicant to an electronic spreadsheet.

### Statistical Analysis

We evaluated MMI scores based on the racial and ethnic group categorization of the applicants. We used descriptive statistics to analyze data for each subgroup and across subgroups of applicants. We compared differences in MMI scores based on age, disadvantaged status, and gender by each racial and ethnic group. The categories we used for this study are listed subsequently. Age categories were 23 years or younger and 24 years or older (age 23 was the median). Race categories were American Indian or Alaskan Native, Asian, Black or African American, Native Hawaiian or Pacific Islander, White, or other. Other includes any racial category reported by applicants apart from the categories otherwise specifically indicated. Ethnic categories were Hispanic or Latino or not Hispanic or Latino. Gender categories were man, woman, or another gender identity. Socioeconomic categories were disadvantaged or not disadvantaged

To conduct bivariate comparisons, we used *t* tests to compare means of the MMI scores across the various groups. We used multiple linear regression to analyze how independent demographic variables affected the MMI score, including covariates for age, gender, and disadvantaged status. We used α at .05 for statistical significance and 2-tailed tests. For the subgroups that demonstrated differences, we also tested for interaction effects that might have yielded inferences on combinations of subgroups assessed using the MMI. We used Stata 18/SE (StataCorp) for data compilation and analyses.

## Results

This study included 3447 applicants to UI COM who participated in MMIs during the 5 interview cycles between 2020 to 2024. Data include distribution by race (56 [2%] American Indian or Alaskan Native, 853 [25%] Asian, 659 [19%] Black or African American, 31 [1%] Native Hawaiian or Pacific Islander; 1502 [44%] White, and 202 [6%] other), ethnicity (660 [19%] Hispanic or Latino; 2787 [80.9%] not Hispanic or Latino), age (1968 [57%] ≤23 years and 1479 [43%] ≥24 years), gender (1518 [44%] men, 1798 [52%] women, and 32 [1%] another gender identity); and disadvantaged status (1198 [35%] with disadvantaged socioeconomic status). The demographic data are summarized in Table 1. Table 2 summarizes gender, age, and disadvantage status by race and ethnicity. We performed the 2-sample *t* test to compare MMI performance by various demographic categories. The results are shown in Table 3. The mean MMI (SD) score for the entire cohort was 3.77 (0.43). One racial category showed statistically significant higher mean MMI scores compared with other applicants: Black or African American (mean [SD], 3.85 [0.44]; *P* < .001). The racial category White showed a statistically significant lower mean (SD) score (3.74 [0.43]; *P* = .001) compared with applicants of other races. There was no statistical difference in mean (SD) MMI in the American Indian or Alaskan Native population (3.73 [0.41]) compared with other applicants, Native Hawaiian or Pacific Islander (3.84 [0.40]) compared with other applicants, or other races (3.75 [0.43]) compared with other applicants. There was also no statistical difference for the ethnic category Hispanic or Latino (mean [SD] MMI, 3.77 [0.45]) compared with other applicants. Younger age (mean [SD] MMI, 3.77 [0.43]) was also not associated with a statistical difference in MMI performance. The gender identity woman (mean [SD] MMI, 3.82 [0.41]; *P* < .001) was associated with a statistically significant higher MMI score, while the gender identity man (mean [SD] MMI, 3.72 [0.45]; *P* < .001) was associated with a statistically significant lower MMI score. There was no statistically significant difference for the category another gender identity (mean [SD] MMI, 3.82 [0.37]). The category disadvantaged (mean [SD] MMI, 3.74 [0.43]; *P* = .009) was associated with statistically significant lower MMI scores compared with other applicants.

**Table 1.** Demographic Characteristics of 3447 Applicants to the University of Illinois College of Medicine Who Participated in the Mulitple Mini Interview, 2020 to 2024

Characteristic	Participants, No. (%)
Race	
American Indian or Alaskan Native	56 **(**1.6)
Asian	853 (24.8
Black or African American	659 (19.1)
Native Hawaiian or Pacific Islander	31 (0.9)
White	1502 (43.6)
Other^a^	202 (5.9)
Not disclosed	144 (4.2)
Ethnicity	
Hispanic or Latino	660 (19.2)
Not Hispanic or Latino	2787 (80.9)
Age, y	
≤23	1968 (57.1)
≥24	1479 (42.9)
Gender identity	
Man	1518 (44.0)
Woman	1798 (52.2)
Another gender identity	32 (0.9)
Not disclosed	99 (2.9)
Disadvantaged status	
Disadvantaged	1198 (34.8)
Not disadvantaged	2249 (65.3)

^a^ Other includes any racial category reported by applicants apart from the categories otherwise specifically indicated.

**Table 2.** Gender Identity, Age, and Disadvantage Status by Race and Ethnicity of 3447 Applicants to the University of Illinois College of Medicine Who Participated in the Mulitple Mini Interview, 2020 to 2024

Race and ethnicity	Total No.	Participants, No. (%)					
		Man	Woman	Another gender identity	Age ≤23	Age ≥24	Disadvantaged
Race							
American Indian or Native Hawaiian	56	26 (46.4)	27 (48.2)	3 (5.4)	31 (55.4)	25 (44.6)	26 (46.4)
Asian	853	362 (42.4)	457 (53.6)	6 (0.7)	558 (65.4)	295 (34.6)	218 (25.6)
Black or African American	659	244 (37.0)	392 (59.5)	2 (0.3)	343 (52.0)	316 (48.0)	339 (51.4)
Native Hawaiian or Pacific Islander	31	10 (32.3)	19 (61.3)	1 (3.2)	14 (45.2)	17 (54.8)	9 (29.0)
White	1502	693 (46.1)	763 (50.8)	19 (1.3)	847 (56.4)	655 (43.6)	384 (25.6)
Other	202	103 (51.0)	91 (45.0)	4 (2.0)	106 (52.5)	96 (47.5)	73 (36.1)
Hispanic or Latino ethnicity	660	301 (45.6)	341 (51.7)	5 (0.8)	373 (56.5)	287 (43.5)	327 (49.5)

**Table 3.** Mean Mulitple Mini Interview (MMI) Scores of 3447 Applicants to the University of Illinois College of Medicine Who Participated in MMI, 2020 to 2024

Characteristic	Mean MMI (SD)	*P* value^a^
Race		
American Indian or Alaskan Native	3.73 (0.41)	.51
Asian	3.77 (0.42)	.98
Black or African American^b^	3.85 (0.44)	<.001
Native Hawaiian or Pacific Islander	3.84 (0.40)	.34
White^c^	3.74 (0.43)	.001
Other^d^	3.75 (0.43)	.57
Ethnicity		
Hispanic or Latino	3.77 (0.45)	.68
Not Hispanic or Latino	3.77 (0.45)	
Age, y		
≤23	3.77 (0.43)	.68
≥24	3.77 (0.43)	
Gender identity		
Man^c^	3.72 (0.45)	<.001
Woman^b^	3.82 (0.41)	<.001
Another gender identity	3.82 (0.37)	.51
Socioeconomic status		
Disadvantaged^c^	3.74 (0.43)	.009
Not disadvantaged	3.78 (0.43)	
Application year (total No. interviewed)		
2020 (613)	3.85 (0.49)	<.001
2021 (756)	3.98 (0.40)	
2022 (698)	3.68 (0.43)	
2023 (687)	3.70 (0.35)	
2024 (693)	3.61 (0.35)	
All years combined (3447)	3.77 (0.43)	

^a^ Two-sample *t* test.

^b^ Statistically significant higher MMI score.

^c^ Statistically significant lower MMI score.

^d^ Other includes any racial category reported by applicants apart from the categories otherwise specifically indicated.

We performed regression analysis for each racial and ethnic category controlling for age, gender, and disadvantaged status. The results are shown in Table 4. For the American Indian or Alaskan Native race category, there was a statistically significant association with higher MMI scores for younger age (coefficient [C] = 0.23; SE, 0.11; *P* = .045) and no association with gender or disadvantaged status. For the Asian race category, there were statistically significant associations with lower MMI scores for men (C = −0.06; SE, 0.03; *P* = .02) and disadvantaged (C = −0.07; SE, 0.03; *P* = .040) subcategories and no association with age (C = 0.05; SE, 0.03; *P* = .12). For the Black or African American race category, there were statistically significant associations with lower MMI scores for the younger age (C = −0.08; SE, 0.03; *P* = .03) and men (C = −0.09; SE, 0.03; *P* = .009) subcategories, and no association with disadvantaged status (C = −0.02; SE, 0.03). For the Native Hawaiian or Pacific Islander race category, there were no associations with gender, age, or disadvantaged status. For the White race category, there were statistically significant associations with lower MMI scores for men (C = −0.97; SE, 0.02; *P* < .001) and disadvantaged (C = −0.68; SE, 0.02; *P* = .007) subcategories. The category other race showed no associations with age (C = −0.10; SE, 0.06), gender (C = −0.10; SE, 0.06), or disadvantaged status (C = −0.69; SE, 0.05). For the Hispanic or Latino ethnic category, there was a statistically significant association with lower MMI score for the men subcategory (C = −0.11; SE, 0.03; *P* = .002) and no associations with age (C = −0.02; SE, 0.04) or disadvantaged (C = −0.06; SE, 0.04) categories.

**Table 4.** Regression Analysis of 3447 Applicants to the University of Illinois College of Medicine Who Participated in Mulitple Mini Interview (MMI), 2020-2024

Race and variable	Coefficient	Standardized effect size	SE	*P* value
American Indian or Alsakan Native				
Aged ≤23 y^a^	0.23	0.53	0.11	.045
Man	−0.06	−0.14	0.11	.61
Disadvantaged status	0.21	0.49	0.11	.07
Asian				
Aged ≤23 y	0.05	0.12	0.03	.12
Man^b^	−0.06	−0.14	0.03	.02
Disadvantaged status^b^	−0.07	−0.16	0.03	.04
Black or African American				
Aged ≤23 y^b^	−0.08	−0.19	0.03	.03
Man^b^	−0.09	−0.21	0.04	.009
Disadvantaged status	−0.02	−0.05	0.03	.55
Native Hawaiian or Pacific Islander				
Aged ≤23 y	−0.24	−0.56	0.14	.10
Man	−0.15	−0.35	0.15	.35
Disadvantaged status	−0.22	−0.51	0.16	.18
White				
Aged ≤23 y	−0.08	−0.19	0.02	.71
Man^b^	−0.97	−2.26	0.02	<.001
Disadvantaged status^b^	−0.68	−1.58	0.02	.007
Other^c^				
Aged ≤23 y	−0.10	−0.23	0.06	.11
Man	−0.10	−0.23	0.06	.08
Disadvantaged status	−0.69	−1.60	0.06	.27
Hispanic or Latino				
Aged ≤23 y	−0.02	−0.05	0.04	.67
Man^b^	−0.11	−0.26	0.03	.002
Disadvantaged status	−0.06	−0.14	0.04	.10

^a^ Statistically significant higher.

^b^ Statistically significant lower.

^c^ Other includes any racial category reported by applicants apart from the categories otherwise specifically indicated.

## Discussion

In this study of MMI performance by a diverse cohort of applicants to a large, public medical school, there were differences in MMI scores by various sociodemographic groups. The breadth of applicant diversity represented in this cohort is noteworthy in comparison with prior MMI research, which has typically been conducted within single institutions or relatively homogeneous applicant pools and has reported limited sociodemographic detail beyond age, gender, race, or broad underrepresented status. Published studies of medical school MMIs have largely focused on reliability and predictive validity rather than examining performance across multiple intersecting demographic characteristics, leaving important gaps in understanding how MMI outcomes vary within highly diverse public-school applicant populations.^3,11^ By contrast, the present study evaluates MMI performance across a wider set of demographic indicators within a large public-sector applicant pool, contributing evidence that is still relatively sparse in the admissions literature.

Two previous studies^5,6^ show that older age and being a woman were associated with better MMI performance, and that disadvantaged status was associated with lower MMI performance. Those same 2 studies,^5,6^ along with an additional study,^7^ showed no difference in MMI performance by applicants identified as underrepresented in medicine. None of those studies investigated whether gender identity, age, or disadvantaged status showed differences among various racial and ethnic statuses.

When all racial and ethnic statuses are aggregated, this study showed that the gender category woman was associated with better MMI performance and disadvantaged status was associated with lower MMI performance. This is consistent with previous studies. This study found no difference in MMI performance based on age, which represents a departure from previous studies. When evaluating MMI performance based on racial and ethnic status, Black or African American applicants scored higher on MMI while White applicants performed lower. There was no difference in performance based on ethnicity.

The regression analysis provides additional insight. For American Indian and Alaskan Native applicants, younger age was associated with higher MMI performance. For Asian applicants, male and disadvantaged statuses were associated with lower MMI performance. For Black or African American applicants, younger age, and being a man were associated with lower performance, but disadvantaged status was not. There was an association with lower MMI scores for White applicants who were men or disadvantaged. Finally, Hispanic or Latino applicants showed an association with lower MMI for those who identify as men.

Several findings in this study are notable. This study did not find a difference in MMI performance based on age when considering the cohort as a whole. Previous studies, which did not investigate individual racial and ethnic categories, showed that older age was associated with higher MMI performance. In this study, younger age was associated with lower MMI performance for Black or African American applicants and better performance for American Indian or Alaskan Native applicants. There were no other associations with age by racial or ethnic category. This suggests that the influence age has on MMI performance is different among different racial and ethnic groups. The overall diversity of this study cohort likely explains why the association of age with MMI performance is different compared with what has been noted in previous studies.

Previous studies showed that disadvantaged status was associated with lower MMI performance.^5,6,7^ This study demonstrated the same for the aggregated cohort. When the various subgroups were disaggregated and evaluated, we noted differences. When considering disadvantaged status by race and ethnicity, this study showed that disadvantaged status is associated with lower MMI performance only for Asian and White applicants. Black or African American, American Indian or Alaskan Native, Native Hawaiian or Pacific Islander, and Hispanic or Latino applicants did not see lower performance based on disadvantaged status. This finding suggests that the experience of being disadvantaged affects racial and ethnic groups differently in relation to performance on MMI.

The gender identity of man was associated with lower MMI performance for each racial and ethnic group in this study except for applicants who identified as American Indian or Alaskan Native and Native Hawaiian or Pacific Islander. For applicants who identify as American Indian or Alaskan Native, younger applicants scored better. With the exception of the applicants who identify as American Indian or Alaskan Native and Native Hawaiian or Pacific Islander, this study is consistent with other studies showing that performance on MMI by male applicants is lower.

This study provides insight into a better understanding of MMI performance by various sociodemographic groups. The MMI is used as an interview format in medical school interviews because it provides a more objective evaluation of a candidate and is less susceptible to bias.^3^ Additionally, MMI can provide for an evaluation of applicant characteristics that are difficult to measure otherwise. For example, MCAT scores and GPAs can measure academic readiness but cannot measure characteristics such as empathy or accountability. Medical school admissions committees develop procedures and strategies for evaluating applicants and can decide how much weight to give to any specific component of an application, including academic metrics and interviews. Ideally, this would be done in a holistic manner with a focus on equity. In deciding how much weight to give to any particular component of an applicant’s evaluation, it is important to have an understanding of the benefits and limits of that component. To that end, understanding that there can be differences in MMI performance based on various sociodemographic characteristics is as important as understanding the benefits and limits of an MCAT score, a GPA, or any other evaluation measure. This study adds to the discourse on the role MMIs have in the evaluation of medical school applicants. Understanding MMI performance may be more complex than reported by prior studies and is worthy of further study.

This study shows that there are differences in MMI performance among various sociodemographic groups; however, this study was not designed to assess why those differences exist. This study was not designed to assess MMI performance of the various sociodemographic groups compared with academic or professional performance in medical school. This study did not assess MMI performance compared with the likelihood of receiving an offer of acceptance to medical school. Additionally, this study did not assess performance on any single MMI station or attribute tested by the individual MMI stations. This study focused solely on the mean MMI performance of the various sociodemographic groups.

The results of this study generate additional research questions regarding the MMI. This study showed that differences in MMI performance exist among various sociodemographic groups. Further exploration should be conducted to understand why those differences exist. The differences could be related to shared or similar experiences of individuals within the various sociodemographic groups. Additionally, the differences could be related to the characteristics and attributes evaluated by the distinct MMI stations. It is unknown if the difference in performance by the various sociodemographic groups seen in this study would persist if a different set of attributes or characteristics were to have been evaluated by the MMI stations. Finally, it is possible that the sociodemographic characteristics of the MMI assessors influence the MMI performance of the candidates being assessed. These topics represent areas for future research on the MMI.

### Limitations

There are limitations to this study. This is a single-institution study. Despite representing a large and diverse cohort of applicants, the nature of a single-institution study introduces the possibility of limited external validity. Some medical schools have used the MMI for many years; however, UI COM has only used MMI since the 2020 admission cycle. Having only 5 years of data available for analysis could limit the generalizability of the study results. This study relied on information provided by the AMCAS application. The AMCAS application data represent self-reported data by the applicant. This could limit the accuracy of some of the data. Each previous study that evaluated disadvantaged status used different criteria in the definition of disadvantaged. It is possible that the differing study designs are not directly comparable regarding the impact disadvantaged status has on MMI performance.

This study assessed mean MMI performance across the various sociodemographic groups. It did not test performance on individual MMI stations. The MMI stations at UI COM are designed to measure self-awareness, ethical and moral judgment, responsibility, and empathy. The MMI stations used at UI COM are not mapped to these characteristics in isolation. Instead, any given station may measure 2 or more of these attributes. This limits the utility of assessing individual stations and represents a limitation of this study.

This study only evaluated sociodemographic characteristics of applicants. It did not evaluate other features of applicants such as prior experience and course work in communication skills and social sciences, which could potentially have an influence on MMI performance and is a limitation of this study.

This study did not evaluate performance on individual MMI stations. It also did not evaluate the sociodemographic characteristics of the MMI facilitators in relation to the applicants. While the design and conceptual frameworks of MMI attempt to mitigate such potential influence, there remains the possibility that influence could be present. This study was not designed to evaluate this interaction.

## Conclusions

In this study of MMI performance by a diverse cohort of applicants to a large, public medical school, there were differences in MMI scores by various sociodemographic groups, suggesting that MMI performance may be more complex than previously reported. These results can guide the appropriate consideration of MMI results in the holistic evaluation of medical school applicants and guide further investigation regarding MMI performance.

## Notes

Supplement. Data Sharing Statement
