# Supplementary material for: Applicant Demographics and Multiple Mini Interview Performance at a Medical School Over 5 Years
Source: JAMA Netw Open. 2026 Apr 22;9(4):e269595. doi: 10.1001/jamanetworkopen.2026.9595 (PMC13103801; doi:10.1001/jamanetworkopen.2026.9595)
Supplement: Supplement. — Data Sharing Statement [file jamanetwopen-e269595-s001.pdf]

Data Sharing Statement

Thompson. Demographics and Multiple Mini Interview Performance at a Public Medical School Over 5 Years. *JAMA Netw Open*. Published April 22, 2026.
doi:10.1001/jamanetworkopen.2026.9595

Data
Data available: No
